# Supplementary material for: Variations in Antimicrobial Activities of Human Monocyte-Derived Macrophage and Their Associations With Tuberculosis Clinical Manifestations
Source: Front Cell Infect Microbiol. 2020 Oct 23;10:586101. doi: 10.3389/fcimb.2020.586101 (PMC7644444; doi:10.3389/fcimb.2020.586101)
Supplement: Supplementary file 1 [file Data_Sheet_1.docx]

**Table S1. Variation in acidification and proteolysis activities in hMDM from TB uninfected individuals (n=18) in response to IgG, TDM and ß- glucan beads**

| **Acidification** | **30 min** | | | **60 min** | | | **90 min** | | |
| --- | --- | --- | --- | --- | --- | --- | --- | --- | --- |
|  | **Range** | **% CV  (mean±SD)** | **P** | **Range** | **% CV  (mean±SD)** | **P** | **Range** | **% CV  (mean±SD)** | **P** |
| **IgG** | 1.02 -1.08 | 1.69 (1.05±0.02) |  | 1 -1.25 | 6.12 (1.12±0.07) |  | 1.02 -1.26 | 6.36 (1.13±0.07) |  |
| **TDM** | 0.97 -1.08 | 2.55 (1.02±0.03) | 0.21 | 1.01 -1.19 | 5.08 (1.08±0.05) | 0.59 | 1.01 -1.22 | 5.05 (1.1±0.06) | 0.57 |
| **β-glucan** | 1 -1.07 | 1.95 (1.03±0.02) |  | 1.01 -1.17 | 4.87 (1.08±0.05) |  | 1.01 -1.22 | 5.23 (1.11±0.06) |  |
|  |  |  |  |  |  |  |  |  |  |
| **Proteolysis** | **60 min** | | | **120 min** | | | **180 min** | | |
|  | **Range** | **% CV  (mean±SD)** | **P** | **Range** | **% CV  (mean±SD)** | **P** | **Range** | **% CV  (mean±SD)** | **P** |
| **IgG** | 1.34 -3.84 | 32.95 (2.05±0.68) |  | 2.22 -15.25 | 48.72 (5.72±2.79) |  | 2.67 -22.92 | 50.01 (8.46±4.23) |  |
| **TDM** | 1.41 -4.85 | 38.94 (2.12±0.82) | 0.81 | 2.61 -15.61 | 51.36 (5.56±2.85) | 0.98 | 3.27 -21.74 | 48.24 (8.14±3.93) | 0.99 |
| **β-glucan** | 1.31 -4.88 | 38.25 (2.16±0.83) |  | 2.82 -17.3 | 51.6 (6.15±3.17) |  | 3.78 -24.5 | 49.05 (9.07±4.45) |  |

Range, min-max; Mean±SD, Mean±standard deviation; CV, coefficient variation, calculated by percentage of SD divided by mean

P, P value for comparison of coefficient variation in either acidification or proteolytic activity among three ligands

**Table S2. Variation in acidification and proteolysis activities in hMDM from LTB (n=35) in response to IgG, TDM and ß- glucan beads**

| **Acidification** | **30 min** | | | **60 min** | | | **90 min** | | |
| --- | --- | --- | --- | --- | --- | --- | --- | --- | --- |
|  | **Range** | **% CV  (mean±SD)** | **P** | **Range** | **% CV  (mean±SD)** | **P** | **Range** | **% CV  (mean±SD)** | **P** |
| **IgG** | 0.99 -1.13 | 3.16 (1.05±0.03) |  | 1.04 -1.24 | 4.31 (1.13±0.05) |  | 1.03 -1.29 | 5.36 (1.15±0.06) |  |
| **TDM** | 0.97 -1.1 | 3.12 (1.02±0.03) | 0.73 | 1.00 -1.19 | 3.97 (1.09±0.04) | 0.71 | 1.02 -1.23 | 4.53 (1.12±0.05) | 0.58 |
| **β-glucan** | 0.98 -1.1 | 2.80 (1.03±0.03) |  | 1.03 -1.19 | 3.75 (1.10±0.04) |  | 1.04 -1.25 | 4.69 (1.13±0.05) |  |
|  |  |  |  |  |  |  |  |  |  |
| **Proteolysis** | **60 min** | | | **120 min** | | | **180 min** | | |
|  | **Range** | **% CV  (mean±SD)** | **P** | **Range** | **% CV  (mean±SD)** | **P** | **Range** | **% CV  (mean±SD)** | **P** |
| **IgG** | 1.49 -6.08 | 37.84 (2.71±1.02) |  | 4.34 -21.06 | 43.79 (8.73±3.82) |  | 4.34 -21.06 | 40.39 (12.69±5.12) |  |
| **TDM** | 1.45 -5.91 | 36.53 (2.71±0.99) | 0.77 | 2.80 -19.38 | 42.87 (8.00±3.43) | 0.95 | 3.53 -24.57 | 39.98 (11.42±4.57) | 0.99 |
| **β-glucan** | 1.39 -6.81 | 41.83 (2.85±1.19) |  | 4.06 -22.04 | 45.77 (8.98±4.11) |  | 6.24 -29.26 | 41.19 (12.82±5.28) |  |

Range, min-max; Mean±SD, Mean±standard deviation; CV, coefficient variation, calculated by percentage of SD divided by mean

P, P value for comparison of coefficient variation in either acidification or proteolytic activity among three ligands

**Table S3. Variation in acidification and proteolysis activities in hMDM from TB uninfected volunteers (n=18) and LTB (n=35) in response to TDM beads**

| **Acidification** | **30 min** | | | **60 min** | | | **90 min** | | | |
| --- | --- | --- | --- | --- | --- | --- | --- | --- | --- | --- |
|  | **Range** | **% CV  (mean±SD)** | **P** | **Range** | **% CV  (mean±SD)** | **P** | **Range** | **% CV  (mean±SD)** | **P** |  |
| **UI** | 0.97 -1.08 | 2.55 (1.02±0.03) | 2.4911E-05 | 1.01 -1.19 | 5.08 (1.08±0.05) | 2.4331E-05 | 1.01 -1.22 | 5.05 (1.10±0.06) | 2.2403E-05 |  |
| **LTB** | 0.97 -1.10 | 3.12 (1.02±0.03) |  | 1.00 -1.19 | 3.97 (1.09±0.04) |  | 1.02 -1.23 | 4.53 (1.12±0.05) |  |  |
|  |  |  |  |  |  |  |  |  |  |  |
| **Proteolysis** | **60 min** | | | **120 min** | | | **180 min** | | | |
|  | **Range** | **% CV  (mean±SD)** | **P** | **Range** | **% CV  (mean±SD)** | **P** | **Range** | **% CV  (mean±SD)** | **P** |  |
| **UI** | 1.41 -4.85 | 38.94 (2.12±0.82) | 1.73E-04 | 2.61 -15.61 | 51.36 (5.56±2.85) | 2.10E-04 | 3.27 -21.74 | 48.24 (8.14±3.93) | 1.71E-04 |  |
| **LTB** | 1.45 -5.91 | 36.53 (2.71±0.99) |  | 2.80 -19.38 | 42.87 (8.00±3.43) |  | 3.53 -24.57 | 39.98 (11.42±4.57) |  |  |

UI, TB uninfected individuals; LTB, Latent tuberculosis

Range, min-max; Mean±SD, Mean±standard deviation; CV, coefficient variation, calculated by percentage of SD divided by mean

P, P value for comparison of coefficient variation in either acidification or proteolytic activity among two groups

**Table S4. Variation in acidification and proteolytic activities in hMDM from UI (n=18) and ATB (n=127) in response to TDM beads**

| **Acidification** | **30 min** | | | **60 min** | | | **90 min** | | | |
| --- | --- | --- | --- | --- | --- | --- | --- | --- | --- | --- |
|  | **Range** | **% CV  (mean±SD)** | **P** | **Range** | **% CV  (mean±SD)** | **P** | **Range** | **% CV  (mean±SD)** | **P** |  |
| **UI** | 0.97 -1.08 | 2.55 (1.02±0.03) | 0.015 | 1.01 -1.19 | 5.08 (1.08±0.05) | 0.015 | 1.01 -1.22 | 5.05 (1.10±0.06) | 0.015 |  |
| **ATB** | 0.98-1.10 | 2.75 (1.03±0.03) |  | 1.00-1.23 | 4.52 (1.1±0.05) |  | 1.00-1.32 | 5.14 (1.14±0.06) |  |  |
|  |  |  |  |  |  |  |  |  |  |  |
| **Proteolysis** | **60 min** | | | **120 min** | | | **180 min** | | | |
|  | **Range** | **% CV  (mean±SD)** | **P** | **Range** | **% CV  (mean±SD)** | **P** | **Range** | **% CV  (mean±SD)** | **P** |  |
| **UI** | 1.41 -4.85 | 38.94 (2.12±0.82) | 0.03 | 2.61 -15.61 | 51.36 (5.56±2.85) | 0.031 | 3.27 -21.74 | 48.24 (8.14±3.93) | 0.027 |  |
| **ATB** | 1.00-4.85 | 30.61 (2.91±0.89) |  | 1.74-15.3 | 32.95 (8.56±2.82) |  | 2.31-21.27 | 29.1 (12.12±3.53) |  |  |

UI, TB uninfected individuals; ATB, Patients with active tuberculosis

Range, min-max; Mean±SD, Mean±standard deviation; CV, coefficient variation, calculated by percentage of SD divided by mean

P, P value for comparison of coefficient variation in either acidification or proteolytic activity among two groups

**Figure S1. Antimicrobial activities of macrophages treated with different ligand beads from healthy volunteers with either uninfected TB or LTB**.

hMDM from uninfected TB (n=18) and LTB (n=35) at day 7 were treated with beads coated with either IgG, TDM or β-glucan to measure acidification activity for 90 min or proteolytic activity for 180 min. The acidification activity index of hMDM from uninfected TB (A) or LTB (C) at 30, 60, and 90 min were plotted. The proteolysis activity index of hMDM from uninfected individuals (B) or LTB (D) at 60, 120, and 180 min were plotted. Box plots represent the interquartile range (IQR) and median. Vertical lines above and below each box extend to the most extreme data point that is within 1.5x IQR. Each dot represents the activity index of hMDM from each subject. P values were determined by Mann-Whitney U test.

**Figure S2. Kinetics of acidification and proteolysis in macrophages from LTB and ATB participants.**

hMDM from LTB (n=35) and ATB (n=101) participants at day 7 were treated with TDM-coated beads to measure acidification for 90 min (A) or proteolysis for 180 min (B). The line and the shaded area represent the mean activities and 95% confidence interval.

**Figure S3. Antimicrobial activities of macrophages from uninfected individuals versus LTB or ATB participants.**

hMDM from uninfected individulas (UI, n=18), LTB (n=35) and ATB (n=101) at day 7 were treated with beads coated with TDM to measure acidification activity for 90 min or proteolytic activity for 180 min. (A) The acidification activity index of hMDM at 30, 60, and 90 min. (B) The proteolysis activity index of hMDM at 60, 120, and 180 min. Box plots represent the interquartile range (IQR) and median. Vertical lines above and below each box extend to the most extreme data point that is within 1.5x IQR. Each dot represents the activity index of hMDM from each subject. P values were determined by Mann-Whitney U test.


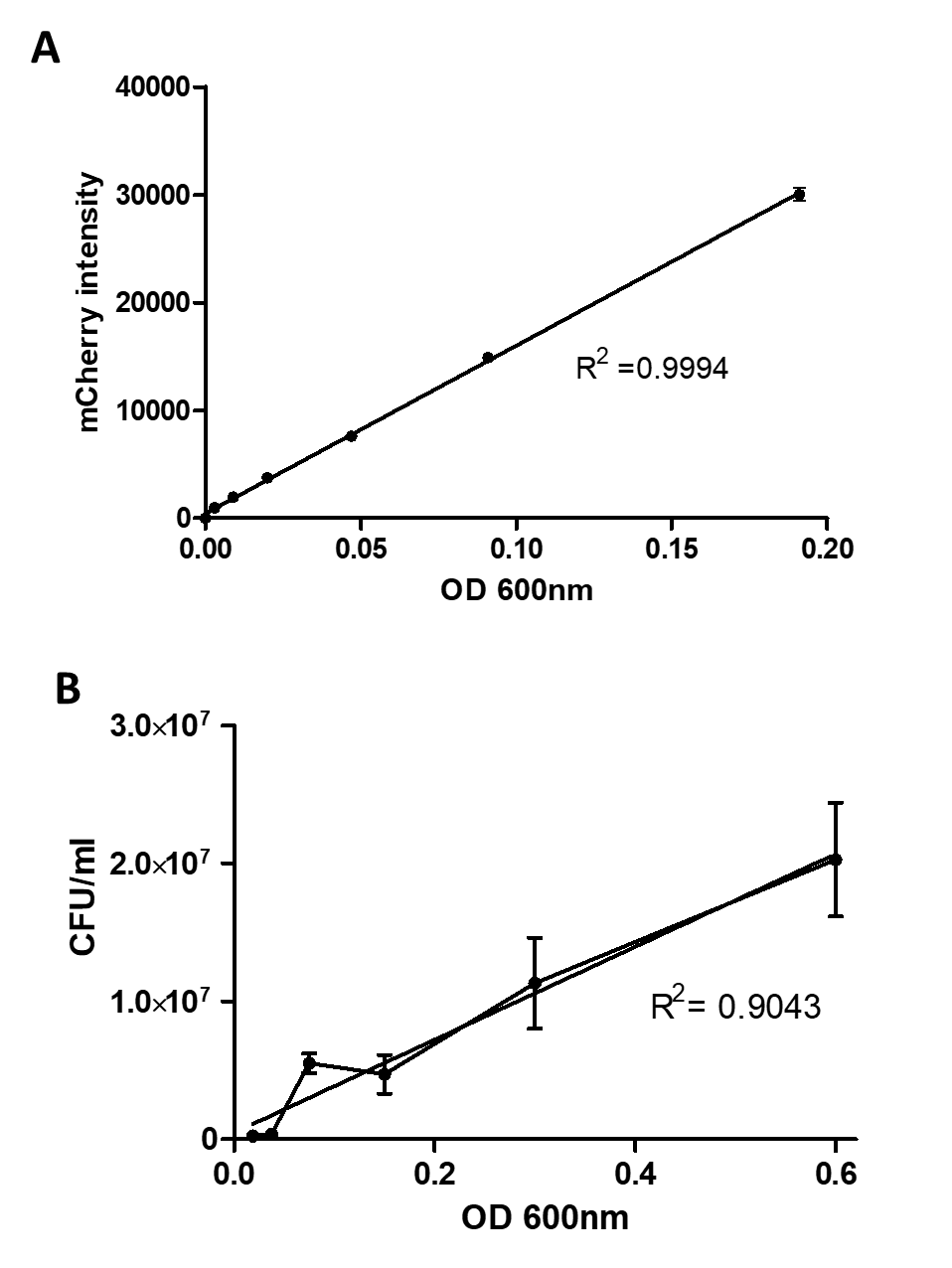


**Figure S4. Correlation of mCherry intensity and bacterial viability**.

(A) Correlation of mCherry fluorescent intensity and bacterial number. Bacteria cultures at an OD 600nm of 0.20 were serially 2-fold diluted. Corresponding fluorescence of mCherry was measured at 620 nm when excited at 575 nm by microplate plate reader. (B) Correlation of optical density and number of viable bacteria. The cultures were serially diluted 10-fold (from 10^-1^ to 10^-4^), sequentially followed by 2-fold serially dilution (from 2^-1^ to 2^-8^) and plated to count the number of CFU. The graph represents the number of CFU in dilution from 10^-4^ to 2^-8^x10^-4^ multiplied with 10^4^, corresponding to adjusted OD 600nm of 0.6, 0.3, 0.15, 0.08, 0.04 and 0.02. The CFU in some first dilutions were uncountable. Data are mean values ± SD for triplicated wells.
